# The Effect of rhCygb on CCl_4_-Induced Hepatic Fibrogenesis in Rat

**DOI:** 10.1038/srep23508

**Published:** 2016-03-23

**Authors:** Zhen Li, Wei Wei, Bohong Chen, Gaotai Cai, Xin Li, Ping Wang, Jinping Tang, Wenqi Dong

**Affiliations:** 1 School of Biotechnology, Southern Medical University, Guangzhou, Guangdong Province 510515, P.R. China

## Abstract

This study aims to investigate whether the use of recombinant human cytoglobin (rhCygb) impact on hepatic fibrogenesis caused by CCl_4_. SD (n = 150) rats were randomly divided into three groups of normal, CCl_4_ model and rhCygb groups. After model establishment, rats in rhCygb groups were administered daily with rhCygb (2 mg/kg, s.c.). Histological lesions were staged according to metavir. Serum parameters including ALT, AST, HA, LN, Col III and Col IV were determined. The liver proteins were separated by 2-DE and identified. As a result, the stage of hepatic damage and liver fibrosis in rhCygb groups were significantly milder than that in CCl_4_ model groups. Meanwhile, rhCygb dramatically reversed serum levels of ALT and AST, and also markedly decreased the liver fibrosis markers levels of LN, HA, Col III and Col IV. In 2-DE, 33 proteins among three groups with the same changing tendency in normal and rhCygb treated groups compared with CCl_4_ model group were identified. GO analysis showed that several identified proteins involved in oxidative stress pathway. The study provides new insights and data for administration of rhCygb reversing CCl_4_-induced liver fibrosis suggesting that rhCygb might be used in the treatment of liver fibrosis.

Liver fibrosis, a pathological process common to chronic liver diseases, is caused by a range of actors, and has a highly deleterious impact on human health. It is well-established that liver fibrosis formation results from an excessive accumulation of extracellular matrix proteins including collagen, a process that occurs in most types of chronic liver disease^1^. Liver fibrosis is described in several stages, a normal liver is at stage 0 to 1, stage 2 represents light or minimal fibrosis, stage 3 is severe fibrosis, and stage 4 denotes advanced liver fibrosis resulting in cirrhosis, liver failure, and portal hypertension^2^,^3^. Any reversibility of advanced liver fibrosis prior to cirrhosis in patients would be of signal importance.

Because of the urgent clinical need, the development of an anti-fibrotic drug would be an auspicious moment in medical research. Up to now, several candidate treatments with anti-fibrotic properties have been developed, which variously act by blocking inflammatory pathways^4^,^5^, inhibiting profibrotic growth factors^6^,^7^,^8^, modulating epigenetic codes^9^,^10^, and interfering with morphogenic pathways^11^,^12^, and which have proven efficacy and tolerability in pre-clinical fibrosis models. However, they are not available to treat fibrotic diseases in clinical practice.

The Cygb protein was first discovered in 2001 by proteomic analysis in rat stellate cells, and was initially named stellate cell-activated protein (STAP)^13^. The protein sequence showed that Cygb was hexacoordinate hemoglobin, representing the fourth member of the globin superfamily in mammals^14^. Furthermore, immunohistochemical analysis revealed that Cygb was ubiquitously expressed in several organs, such as kidney and pancreas, and localized in fibroblast-like cells^15^. Cygb was a 21.4 kDa protein consisting of 190 amino acids and mapped at the 17q25.3 chromosomal segment. Exogenous over-expression of Cygb was soon demonstrated experimentally, both *in vitro* and *in vivo*, to protect hepatic stellate cells (HSC) from oxidative stress and suppress their differentiation to a myofibroblast-like phenotype^16^,^17^,^18^. Further, it was reported that Cygb had active peroxidase properties, which protected cells against oxidative damage by free radicals^19^,^20^,^21^. These findings prompted the hypothesis that Cygb could be effective in the prevention or treatment of liver fibrosis. In this study, therefore, we investigate the effect of rhCygb on CCl_4_-induced liver fibrosis in rats at various stages of the disease’s progression, from mild to severe fibrosis.

## Materials and Methods

### Preparation of rhCygb

Human cytoglobin cDNA was obtained from HepG2 cells by RT-PCR. A recombinant construct of pET28a-hCyt were transformed into *E. coli* BL21 (DE3). For expression, the bacteria cultures were induced for 4 h using 1 mM isopropyl β-d-thiogalactoside (IPTG) after the culture OD 600 has reached 0.6. The total bacteria cells were collected by centrifugation at 5,000 rpm for 10 min, resuspended in PBS, and broken down by sonication. The rhCygb was purified by sephadex G-25 and affinity chromatography which the monoclonal antibody was prepared by our laboratory. The purified protein was analyzed with SDS–PAGE and identified by mass spectrometry with ABI 4700 Proteomics Analyzer (Applied Biosystems, USA). The yields of rhCygb measured by the software BandScan 5.0 based on SDS-PAGE results. The total antioxidation capacity (T-AOC) of rhCygb was measured spectrophotometrically as described by Tang *et al.*^22^, using a T-AOC commercial kits (Jiancheng, Nanjing, China) according to the guidelines of the kit.

### Ethics statement

All experimental procedures were conducted in conformity with institutional guidelines approved by the Chinese Association of Laboratory Animal Care. All experimental protocols were approved by the Institutional Animal Care and Use Committee in Southern Medical University, Guangzhou, China.

### Animals

Sexually mature sprague–dawley (SD) rats (n = 150) weighing 180–220 g were obtained from the experimental animal center of Southern Medical University. Care of the animals used in this investigation was conducted according to the guidelines approved by the Chinese Association of Laboratory Animal Care. Rats were housed, five per cage, under constant temperature (20 ± 2 °C) and humidity (70%) with a 12 h light/dark cycle and with free access to chow and water.

### Acute toxicity test

BALB/c mice of both sexes, weighing 18–24 g, were obtained from the experimental animal center of Southern Medical University. The acute toxicity of rhCygb was determined *in vivo* using the procedure approved by Chen *et al.*^23^. Uninfected mice were peritoneal injected rhCygb at doses of 100 and 500 mg/kg body weight. The animals were monitored for any toxic symptoms or mortality over a 14-day period.

### Induction of fibrosis

Three degrees of liver fibrosis were induced by chronic carbon tetrachloride (CCl_4_) administration at three different dosage levels:

- Mild model group: 2 ml/kg of 25% CCl_4_ in paraffin oil (Sigma Co., Milan, Italy) was administered by subcutaneous injection twice a week (Tuesday and Friday) for 10 weeks;
- Medium model group: treatment in group (a) plus 50% CCl_4_ treatment for 3 more weeks;
- Severe model group: treatment in group (b) plus 62.5% CCl_4_ for 2 more weeks.

### Experiment design

**Figure 1:**
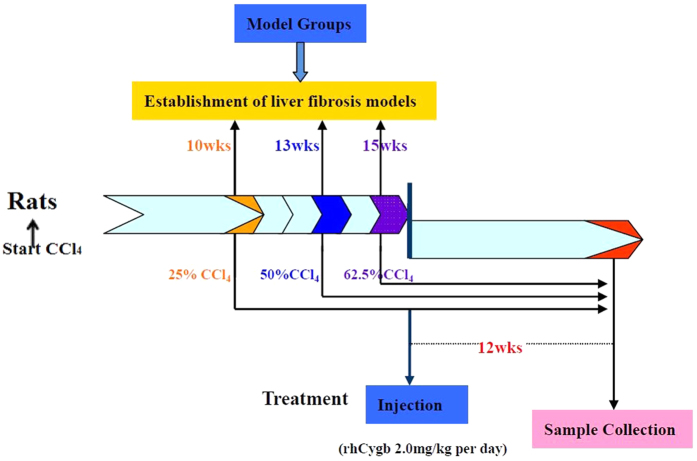
Schedule for the treatment and experimental tests. Rats were injected with CCl_4_ for three different doses to establish mild, medium and severe models at 10, 13 and 15 weeks, 2 times per week. After model was established, rats were treated daily with saline, CCl_4_ or rhCygb + CCl_4_ for 12 weeks. Rats were sacrificed within 24 h after the last treatment.

To evaluate the effect of rhCygb on liver fibrosis, 30 rats were used in each of the groups (group a, b, c). A schedule of the treatment is shown in Fig. 1. In each group, followed by CCl_4_ treatment, rats were further treated with saline (vehicle group), CCl_4_ (model group) or rhCygb (rhCygb group, 2 mg/kg body weight/day, s.c. injection) in combination with CCl_4_ for 12 weeks. At the end of the experiments, all rats were sacrificed within 24 h after the last injection.

### Chemical analysis

Venous blood samples were collected for serum separation. Samples were stored at −20 °C until further analysis. Aspartate aminotransferase (AST) and alanine aminotransferase (ALT) levels were measured spectrophotometrically by Olympus/AU 5200 (Konsesyum, Alternative Biomedical Services, Dallas, TX, USA).

Serum liver fibrosis markers hyaluronic acid (HA), laminin (LN), collagen III (Col III) and collagen IV (Col IV) were determined using enzyme-linked immunosorbent assay (ELISA) kits (SIGMA chemical, Corp.).

### Pathological studies

In the end of the experiments, rats liver were removed and rinsed with cold saline water. Then liver lesions were fixed, embedded, and stained with Sirius red and hematoxylin-eosin (HE)^24^. Their evaluation was conducted using a light microscope (Axiphot2, Carl Zeiss) blindly. Fibrosis was scored according to the Metavir scoring system reported by Bedossa *et al.*^25^. Intralobular degeneration and focal necrosis, portal inflammation and fibrosis were compared across the groups. Three representative images of each histology sample section from each rat were selected randomly and all of the rats were scored.

### Protein separation

Five liver samples (approximately 30–40 mg wet) were removed from vehicle group, CCl_4_ model group and rhCygb treated group (medium). The samples were then frozen in liquid nitrogen and homogenized with a mortar and pestle. Proteins were prepared in lysis buffer. A total of 120 μg of protein was separated by two-dimensional electrophoresis (2-DE) according to the product manual ‘2-DE for Proteomics’ (Bio-Rad, U.S). After silver staining, each spots were cut out from the gel and digested using trypsin according to the method described by Qualtieri *et al.*^26^.

### MS data analysis

Peptide mixtures of each gel spot were dissolved in 0.1% TFA, desalted, and concentrated. The samples were then mixed with the same volume of matrix (CHCA in 30% ACN/0.1% TFA), spotted on a target disk, and allowed to air-dry. Samples were analyzed using a Bruker ultrafleXtreme MALDI-TOF mass spectrometer (Bruker Daltonics, Germany). The protein database search was performed using the MASCOT search engine (http://www.matrixscience.com/)^27^. Mass tolerance was allowed within 150 parts/million (ppm). Proteins matching more than five peptides and with a MASCOT score higher than 60 were considered significant (*p* < 0.05).

### Bioinformatics analysis

To identify significantly represented biological themes and functional groups in the protein list (Table 1), the gene ontology (GO) and pathway analysis were performed using the database for annotation, visualization and integrated discovery (DAVID) program (https://david.ncifcrf.gov/)^27^. The GO analysis was used to identify enriched biological themes by the GeneCodis tool (http://genecodis.cnb.csic.es/)^27^. The list of identified proteins was used as the input data when the DAVID default population background was used; We used EASE scores, which modified Fisher’s exact test P values < 0.05 and corrected for multiple testing by the Benjamin–Hochberg method.

Modified gene set enrichment analysis (GSEA) was used to assess functional significance at the level of sets of genes as previous described by Li *et al.*^28^. All of these potential targets were compared with the “c2_all” collection of curated gene sets from the Molecular Signatures Database (GSEA Version 2.0.14), consisting of 1077 gene sets corresponding to BIOCARTA, KEGG and REACTOME biological pathways. The False Discovery Rate (FDR) q value is deemed significant value at <0.05.

### Statistical analysis

All data were expressed as the mean ± standard deviation from at least 5 independent experiments. Figures were obtained from at least 5 independent experiments with similar patterns. The biochemical experimental data were analyzed by two-way ANOVA. The Kruskal-Wallis was used for comparisons. Statistical analyses were performed using SPSS software version 19.0 (SPSS Inc, Chicago, USA).

## Results

### Purity and activity of rhCygb

The purity of rhCygb was 95% by BandScan 5.0 software. The antioxidant activity of rhCygb is 95.58 ± 2.67 U/mg (*p* < 0.01) (supplemental data).

### Acute toxicity results

When given as a single dose of 100 and 500 mg/kg, rhCygb produced no signs of acute toxicity in the 14-day period of observation. No death was recorded in the 100- and 500-mg/kg-treated group. All groups of mice exhibited stable temperature values throughout the period of observation.

### Developments of CCl_4_-induced hepatic fibrosis in rats

**Figure 2:**
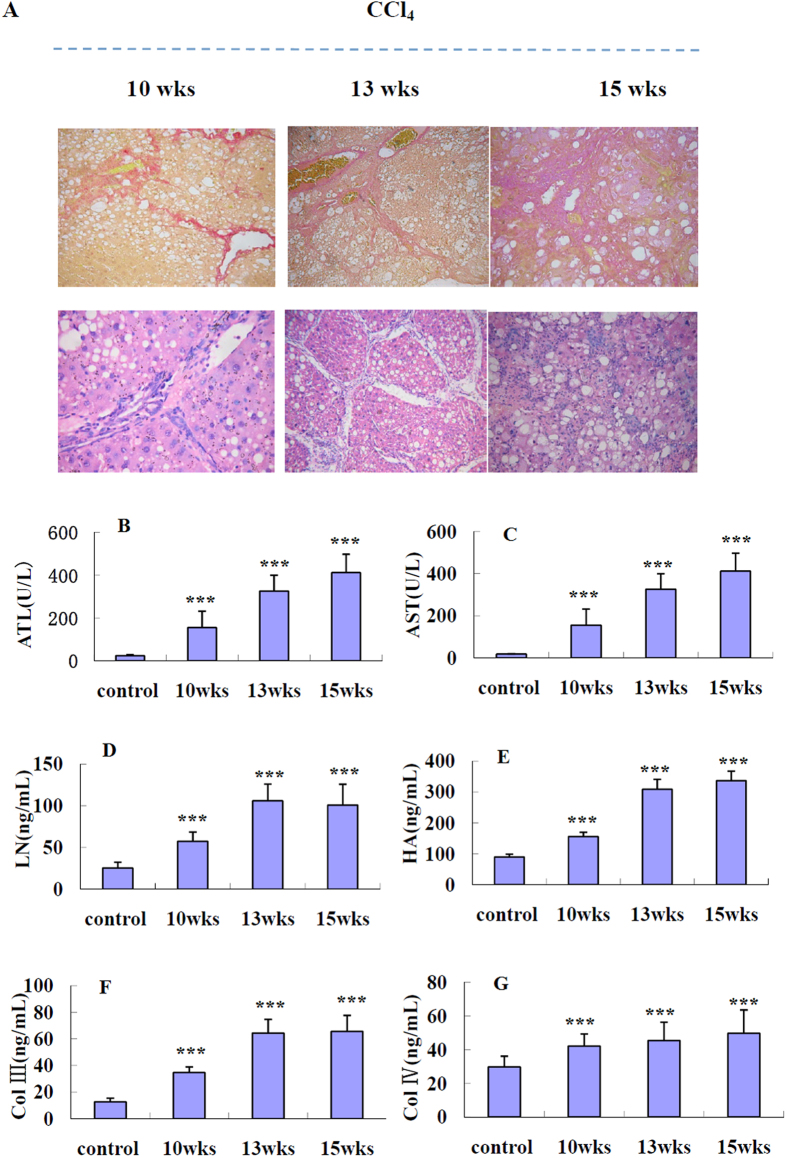
Examination of liver histology and serum biochemistry at different stages (10, 13, 15 weeks) in CCl_4_ model rats. (**A**) Representative photomicrographs of HE and Sirius red staining for observing the morphology of fibrosis of rat livers at 10, 13, 15 weeks (×200); Serum levels of (**B**) ALT, (**C**) AST, (**D**) LN, (**E**) HA, (**F**) Col III and (**G**) Col IV.

All experiments successfully induced fibrosis, as determined by the examination of liver histology and serum biochemistry (Fig. 2A–G). The mortality was 0 in all the experiment groups. According to the results of development of rat liver fibrosis model, rats treated with CCl_4_ were sacrificed at 3 different times, 10th week, 13th week and 15th week. At sacrifice, all rats presented marked fibrosis with significant differences in the amount of liver fibrotic tissue among groups. Hepatolobular injury was observed with centrilobular necrosis, balloon cells, and lipids accumulation in 15-weeks-model rats (Fig. 2A). The same results were obtained in an analysis of serum biochemistry (Fig. 2B–G). At the lowest dose (25% CCl_4_) for 10 weeks in SD rats, administration of CCl_4_ significantly (*p* < 0.01) increased the concentration of AST, ALT, LN, HA, Col III and Col IV in rat serum compared with the levels in the vehicle group (Table 2). Moreover, administration of a higher dose of CCl_4_ (50% CCl_4_) for a further 3 weeks in group b (medium) resulted in a marked prolongation of toxicity, increasing (*p* < 0.01) serum enzyme values about two-fold except for Col IV. However, there was no significant (*p* > 0.05) difference between the group b (medium) and group c (severe).

### Effects of rhCygb on hepatic histoarchitecture

**Figure 3:**
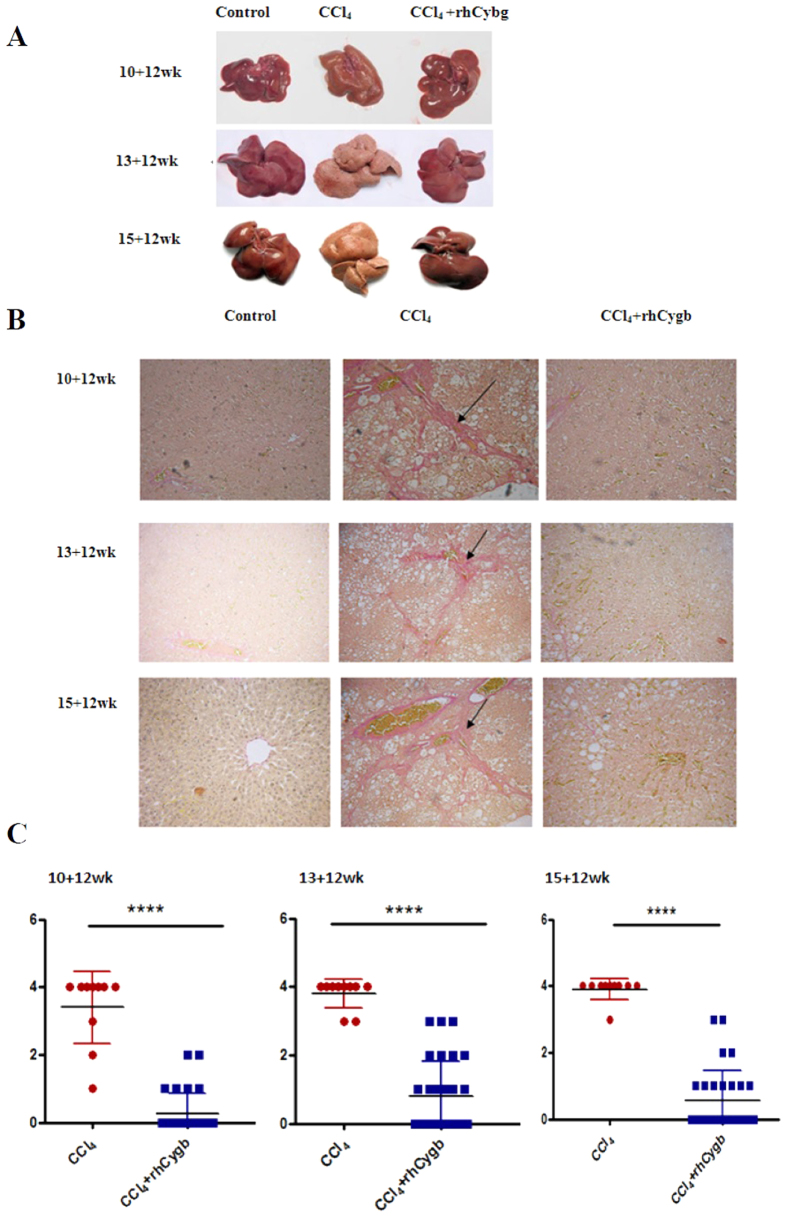
rhCygb treatment attenuates CCl_4_-induced rat liver fibrosis. (**A**) The liver appearance of vehicle control, CCl_4_ model and rhCygb groups. (**B**) Representative photomicrographs of Sirius red staining for observing the morphology of fibrosis of rat livers from different groups (×200). Black arrows indicate fibrosis. (**C**) Fibrosis scores (Y axis) of CCl_4_ model groups (n = 10) and rhCygb treatment groups (n = 30). Fibrosis scores are based on the percentage of liver area positively stained by Sirius red; ****p < 0.001.

We next evaluated the effect of rhCygb on liver fibrosis following chronic CCl_4_ administration. Rats were treated with rhCygb (2 mg/kg/day, s.c.) in combination with CCl_4_ (25%, 50% or 62.5%) for 12 weeks. All of the rats survived during the experimental period. The influence of CCl_4_ and rhCygb on liver was depicted in Fig. 3, in which liver fibrosis was advanced for 12 more weeks. In this group, fibrotic changes caused by CCl_4_ reversed dramatically by rhCygb. As shown in Fig. 3A, the liver appearance of CCl_4_-administered rats significantly changed, especially in the medium and severe groups. These groups, after treatment with rhCygb, exhibited a clear and marked improvement in their liver, when compared with those of the CCl_4_-administered groups. Photomicrographs of hepatic specimens stained with Sirius red were depicted in Fig. 3B, and the scores of fibrosis variables were presented as medians in Fig. 3C. As shown in the figures, no hepatic fibrosis occurred in the control group, which showed an integrated lobular structure with central venous and hepatic cord radiation. The staging score was 0. Extensive fibrosis and ballooning of hepatocytes were observed in all the CCl_4_ model groups. As expected, the high CCl_4_ treatment caused fibrous connective tissue proliferation as well as a good deal of collagen fiber formation. Moreover, increased dosages caused even more frequent pseudo lobules to appear in model control groups. The hepatic fibrosis score in the CCl_4_ groups increased to 3.40 ± 0.33, 3.80 ± 0.13 and 3.90 ± 0.1, respectively. In the rhCygb treated groups, liver had little collagen fiber formation in the peripheral area, and no pseudo lobules. The positive area of Sirius red staining was significantly lower than that of the CCl_4_ groups. The scores were 0.27 ± 0.58 (mild group), 0.8 ± 0.19 (medium group) and 0.57 ± 0.16 (severe group), respectively. Similar observations were made in rhCygb groups which evinced statistically significant lower fibrosis scores compared to CCl_4_ groups.

### Indices of hepatotoxicity: liver marker enzymes

The levels of serum ALT and AST are markers of liver damage. As shown in Table 2, AST and ALT level significantly increased in mild, medium and severe CCl_4_ model groups compared with normal control groups, reflecting hepatocelluar damage in CCl_4_-induced liver fibrosis. Then rhCygb treatment significantly (*p* < 0.01) reversed serum ALT and AST levels in 10 + 12 weeks, 13 + 12 weeks and 15 + 12 weeks groups, to the levels almost equal to vehicle controls.

### Effects of rhCygb on levels of LN, HA, Col III and Col IV

In this study, serum LN, HA, Col III and Col IV levels in CCl_4_ groups were higher (*p* < 0.01) than controls (Table 2). Compared with the CCl_4_ groups, rhCygb also markedly decreased (*p* < 0.01) liver fibrosis marker levels of LN, HA, Col III and Col IV in 10 + 12 weeks, 13 + 12 weeks and 15 + 12 weeks groups.

### Differential 2-DE analysis of liver tissue proteins

**Figure 4:**
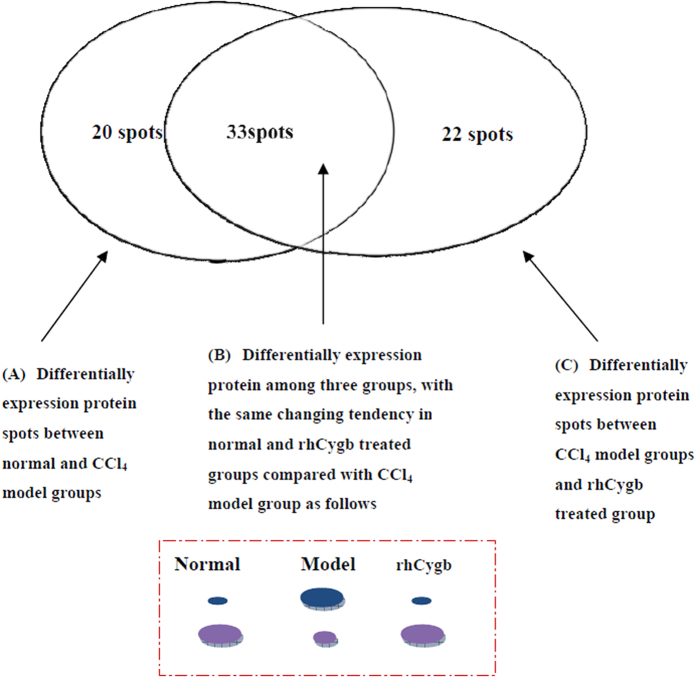
Grouping of differentially expressed protein spots. There were 53 differential spots between vehicle and CCl_4_ model groups (pattern A), 55 differential between model and rhCygb groups (pattern C), and 33 spots were overlapped between pattern A and pattern C, in which vehicle and rhCygb groups had the same tendency of differential expression compared with ones in model group (pattern B).

To study the influence of rhCygb on the proteome of CCl_4_-induced fibrotic liver, liver tissues were harvested from the (13 + 12)-weeks-old control group, the CCl_4_ model group, and rhCygb treatment group of rats. When examined, the 2-DE gel showed an obvious separation of liver tissue proteins, with 1312 proteins in the vehicle group, 1325 proteins in the CCl_4_ model group and 1259 proteins in rhCygb group. Comparing the spots of vehicle to model group or model to rhCygb group by PDquest software for image analysis, a total of 75 protein spots exhibited significant (*p* < 0.05) differences in protein expression between vehicle and model or between model and rhCygb group (supplement data). These 75 protein spots were grouped into three major patterns (Fig. 4): pattern A: 53 spots differentially expressed between vehicle and CCl_4_ group; pattern C: 55 spots differentially expressed between CCl_4_ model and rhCygb group; pattern B: 33 spots which overlapped pattern A and pattern C. For example, the expression of cathepsin D in the vehicle group was lower compared to model, while that in rhCygb treated group was also lower. Among the 33 proteins, the expression of 21 proteins were three times higher and that of 12 proteins was three times lower in the vehicle and rhCygb treated groups than in the CCl_4_ model group significantly (*p* < 0.05). A total of 33 peptide mass fingerprints (PMFs) were matched and identified by MALDI-TOF-MS and protein database searching, as displayed in Table 1.

### Functional annotation of differentially expressed proteins

We analyzed the GO enrichment of these upregulated and downregulated proteins (Table 1). Relevant Protein functions were listed in Table 3. These dysregulated proteins were predicted to be involved in different metabolic processes including the response to stimulus, oxidation-reduction processes, heat-shock protein, the catabolic process and the cytoskeleton.

GSEA (Table 4) revealed that up-regulated genes in rhCygb treated group were significantly enriched in the down-regulated gene set in hepatocellular carcinoma (ranked 1^st^, *p* = 3.33 e^−8^, FDR = 2.27 e^−4^), or in the genes highly expressed in hepatocellular carcinoma with good survival (ranked 4^th^, *p* = 4.19 e^−5^, FDR = 2.77 e^−2^). FDR q-value was considered significant at < 0.05.

## Discussion

Much experimental evidence and clinical studies has shown that the physiological functions of Cygb are associated with various diseases, such as cancer^29^, gastroesophageal reflux disease^30^, psychomotor retardation epilepsy^31^, and certain neurodegenerative disorders^32^, especially liver fibrosis. Hence, the aim of the present research was to study the mechanism of liver fibrosis and rhCygb anti-fibrotic action.

### Effects of rhCygb on serum markers of fibrosis

CCl_4_ proved to be highly useful as an experimental model for the study of liver damage in humans^33^,^34^,^35^. It is usually used through intraperitoneal injection or oral administration, and with a dosage of 0.2 to 5 ml/kg liver fibrosis is achieved between 4 and 18 weeks^36^,^37^. In this study, the mild, medium and severe liver fibrosis rat models were developed through differential doses (25–62.5%) and exposure duration (10–15 weeks) of CCl_4_. The results of the present study revealed that continuous and increasing doses of CCl_4_ resulted in advanced fibrosis which caused an elevation of levels of serum marker enzymes such as ALT, AST, HA, LN, Col III and Col IV from the mild to the severe model. The serum marker enzymes ALT and AST have previously been reported as important diagnostic factors for hepatic diseases, as they are cytosolic enzymes of the hepatocyte, and increased activity in their circulation reflects cell damage and leakage^38^. High ALT and AST levels are associated with a higher risk of fibrosis progression. The present study demonstrates that rhCygb can reduce heightened ALT and AST, indicating that they can promote the repair of injured liver cell.

In addition, HA, LN, Col III and Col IV have been found to be ideal serum markers of hepatic fibrosis, which play an important role in detecting the degree of hepatic fibrosis^39^. In the present study, CCl_4_ model groups increased markedly compared with normal groups. However, after the treatment of rhCygb (therapy but not removing CCl_4_), we detected that the serum contents of HA, LN, Col III and Col IVdecreased to the level of normal groups. This may be the basic mechanism of rhCygb reversing rat hepatic fibrosis.

### Effects of rhCygb on stages of fibrosis

In response to liver injury induced by whatever cause, collagen and matrix proteins are produced in much higher quantities by activated hepatic stellate cells, which ultimately leads to liver fibrosis^40^. Fibrosis is evaluated by using Sirius red staining and scored using the fibrosis staging system (the Metavir score). The Metavir system scores necroinflammatory activity from 0 to 3 and fibrosis from 0 to 4^25^. In this study, evaluation of staging revealed a score of 4 in different cases in CCl_4_ model groups: 70% in the mild group, 80% in the medium group and 90% in the severe group. However, score 4 didn’t show up in any of the rhCygb treated groups. In time, score 0 was present in 24 rats in the mild group, 18 rats in the medium group, and 19 rats in the severe group. These data are in solid agreement with the activities of serum biomarkers. Interestingly, the stages of fibrosis were previously believed to be progressive and largely irreversible. There is now mounting experimental and clinical evidence suggesting that liver fibrosis can regress in all chronic liver diseases by withdrawing the cause of liver injury or by treatment of the disease^41^,^42^. Our paper also obtains that CCl_4_-induced rat liver fibrosis was markedly reversed by administration of rhCygb. He *et al.* reported the similar findings in their use of rhCybg in the thioacetamide (TAA)-induced rat liver fibrosis model^43^.

### Effects of rhCygb on protein species

We systematically analyzed the effect of rhCygb on liver proteome in the CCl_4_-induced rat model. Thirty-three differentially expressed proteins appeared among the three groups, with the same alteration profile in the vehicle and rhCygb treated groups compared with the CCl_4_ model group, identified through comparative proteomics. Functional annotation analysis showed several identified proteins involved in oxidative stress pathways, such as the response to chemical stimuli, oxidative-reduction, and xenobiotic metabolic processes. Oxidative stress is thought to be a critical factor in the development of CCl_4_-induced liver fibrosis^44^. Previous studies have reported the effects of rhCygb on the levels of oxidative stress biomarkers and anti-oxidative capacity^45^. The current results show that rhCygb regulates antioxidant enzymes such as peroxiredoxin-2, glutathione S-transferase, glutathione peroxidase 1, cytochrome P450 2C11, biliverdin reductase A, estrogen sulfotransferase, and taste receptor type 1 member 2. One of these proteins, P450 2C11, an enzyme that was increased by rhCygb, plays an important role in the deactivation of estrogens via 16a-hydroxylation and catechol formation. It is particularly active in relation to the development of fatty change, hepatic fibrosis, portal hypertension, and cirrhosis^46^. Earlier studies have shown that P450 2C11 activity in rat models of experimental liver disease including hepatic cirrhosis is produced by CCl_4_^47^. The present study also demonstrated that the level of protein P450 2C11 in the liver decreased significantly in this model of liver fibrosis at the 25-week time point. In addition, we also found that enzymes involved in fatty acid β-oxidation (Long-chain-fatty-acid-CoA ligase 1, Enoyl-CoA hydratase, Carboxylesterase 1D and ATP synthase sub-unit alpha) were down-regulated in CCl_4_ model group, whereas in the rhCygb-treated group they were up-regulated (as they were in the vehicle group). These findings show that impairment of fatty acid oxidation may contribute to hepatic fibrosis.

In summary, the results of the present study demonstrate that in addition to the effect of reversing liver fibrosis, rhCygb could reduce the serum levels of ALT, AST, HA, LN, Col III and Col IV, indicating that it has definite effects in reversing the formation of liver fibrosis in the course of chronic hepatitis. The differentially expressed proteins have several groups of biological functions, especially relating to oxidative stress, which are helpful in revealing the underlying mechanisms of rhCygb action against liver fibrosis. rhCygb could be a novel candidate drug in combating liver fibrosis.

## Additional Information

**How to cite this article**: Li, Z. *et al.* The Effect of rhCygb on CCl_4_-Induced Hepatic Fibrogenesis in Rat. *Sci. Rep.* **6**, 23508; doi: 10.1038/srep23508 (2016).

## Supplementary Material

Supplementary Information

## Figures and Tables

**Table 1:** Identification of 33 differentially expressed proteins among normal, CCl_4_ model, and rhCygb groups.

Code	Protein name	Swiss-prot Accession	MW	PI	Protein function
5	Cathepsin D↓	Q6P6T6	45107.04	7.11	Proteolysis; Extracellular matrix
7	Pregnancy-specific beta 1-glycoprotein↓	Q9Z1D6	43326.17	8.73	Pregnancy
9	DnaJ homolog subfamily B member 1↓	Q6TUG0	40754.77	6.26	Serves as a co-chaperone for HSPA5
10	Twinfilin-1↓	Q5RJR2	40293.57	6.65	Regulation of actinphosphorylation; Actin cytoskeleton
11	Haptoglobin↓	P06866	39051.73	6.51	Acute inflammatory response; Acute inflammatory response; Response to hypoxia; Organ regeneration
12	Tubulin beta-4B chain↓	Q6P9T8	50225.16	4.52	Major constituent of microtubules; Structural constituent of cytoskeleton
15	Transitional endoplasmic reticulum ATPase↓	P46462	89976.99	4.89	ER to Golgi vesicle-mediated transport; ATP catabolic process
17	Beta-lactamase-like protein 2↓	Q561R9	32748.81	6.29	Hydrolase activity; Metal ion binding
18	Microtubule-actin cross-linking factor 1↓	D3ZHV2	623207.64	5.07	Wound healing; Epidermal cell migration; Regulation of microtubule-based process
20	GTP-binding protein SAR1b↓	Q5HZY2	22509.55	6.02	ER-Golgi transport; Intracellular protein transport
21	Adenine phosphoribosyltransferase↓	P36972	19761.48	6.52	Adenine salvage; Cellular response to insulin stimulus
22	Protein Sar1a↑	Q6AY18	22498.54	6.68	ER-Golgi transport; Vesicle-mediated transport
25	Long-chain-fatty-acid–CoA ligase 1↑	P18163	79154.6	6.98	Fatty acid metabolism; Beta-oxidation
28	Protein Eea1↑	F1LUA1	162091.42	5.54	Endocytosis; Vesicle fusion
29	Cytochrome P450 2C11↑	P08683	57657.6	7.77	Xenobiotic metabolic process; Monooxygenase; Oxidoreductase
31	Estrogen sulfotransferase↑	P49889	35734.66	5.52	Utilizes 3′-phospho-5′-adenylyl sulfate (PAPS) as sulfonate donor
32	Biliverdin reductase A↑	P46844	33715.58	6.01	Oxidoreductase; Biliverdin reductase activity
33	Ketohexokinase↑	Q02974	33298.74	6.65	Carbohydrate catabolic process; Response to insulin
34	Protein Inmt↑	D3ZNJ5	29989.04	5.81	Thioether S-methyltransferaseactivity; Amine N-methyltransferase activity
35	Mitochondrial GTPase 1↑	E9PTB3	36994.54	9.68	Mitochondrial GTPase activity
37	Taste receptor type 1 member 2↑	F8WFI0	62271.78	8.15	Oxidoreductaseactivity; Acting on the aldehyde or oxo group of donors, NAD or NADP as acceptor
39	Putative L-aspartate dehydrogenase↑	G3V9Z4	30654.77	6.84	NAD biosynthetic process; NADP catabolic process
40	Phenazine biosynthesis-like domain-containing protein↑	Q68G31	31952.24	6.25	Biosynthetic process
41	Carbonic anhydrase 3↑	P14141	29697.82	7.4	Liver response to oxidative stress; Carbonate dehydratase activity
42	Enoyl-CoA hydratase, mitochondrial↑	P14604	31895.34	8.27	Lipid metabolism; Fatty acid beta-oxidation
43	Protein Urad↑	M0RCU5	24634.52	6.44	Carboxy-lyase activity; Allantoin biosynthetic process
44	Glutathione S-transferase Yb-3↑	P08009	25835.08	7.43	Cellular detoxification of nitrogen compound; Xenobiotic catabolic process
45	Peroxiredoxin-2↑	P35704	21941.13	5.28	Removal of superoxide radicals; Response to oxidative stress; Peroxidase activity
46	ATP synthase subunit alpha↑	F1LP05	59889.71	9.72	ATP synthesis coupled proton transport; Lipid metabolic process
47	Glutathione peroxidase 1 ↑	P04041	22463.37	8.05	Oxidoreductase; Peroxidase; Cell redox homeostasis; Negative regulation of inflammatory; Response to toxic substance
48	Major urinary protein↑	P02761	21008.61	6.13	Negative regulation of lipid storage; Negative regulation of gluconeogenesis; Positive regulation of lipid metabolic process
49	Carboxylesterase 1D ↑	P16303	62392.85	6.52	Lipid degradation; Lipid metabolism; acylglycerol catabolic process; Major lipase in white adipose tissue
50	Nucleoporin GLE1↑	Q4KLN4	79954.74	7.65	Poly(A) + mRNA export from nucleus; Protein transport

**Table 2:** Effects of rhCygb on ALT, AST, LN, HA, Col III and Col IV of CCl_4_-induced liver injury SD rats ( 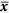 ± SD, n = 10).

	Group	ALT(U/L)	AST(U/L)	LN(ng/ml)	HA(ng/ml)	Col III(ng/ml)	Col IV(ng/ml)
Mild model	control	24.45 ± 4.02	27.37 ± 6.32	29.76 ± 6.32	112.19 ± 16.66	13.09 ± 2.77	28.28 ± 5.44
	CCl_4_	456.37 ± 95.34^a^	588.69 ± 115.75^a^	93.30 ± 11.87^a^	352.64 ± 33.62^a^	78.86 ± 10.18^a^	50.02 ± 5.31^a^
	CCl_4_ + rhCygb	48.32 ± 7.53^c^	52.32 ± 5.32^c^	30.37 ± 3.84^c^	122.79 ± 19.96^c^	19.40 ± 5.47^c^	31.04 ± 5.35^c^
Medium model	control	33.52 ± 4.3	31.58 ± 6.35	28.45 ± 6.35	107.33 ± 15.26	15.3 ± 3.5	29.35 ± 5.23
	CCl_4_	501.46 ± 98.77^a^	642.33 ± 98.77^a^	113.75 ± 12.4^a^	398.95 ± 48.23^a^	88.23 ± 15.6^a^	56.78 ± 7.89^a^
	CCl_4_ + rhCygb	44.25 ± 7.6^c^	45.26 ± 7.6^c^	32.52 ± 7.6^c^	118.56 ± 25.68^c^	16.2 ± 3.3^c^	30.24 ± 5.66^c^
Severe model	control	35.52 ± 4.3	37.58 ± 6.35	26.45 ± 6.35	97.33 ± 15.26	14.3 ± 3.5	26.35 ± 5.23
	CCl_4_	521.46 ± 98.77^a^	622.33 ± 98.77^a^	103.75 ± 12.4^a^	378.95 ± 48.23^a^	78.23 ± 14.6^a^	58.78 ± 7.89^a^
	CCl_4_ + rhCygb	47.25 ± 7.6^c^	55.26 ± 7.6^c^	28.52 ± 7.6^c^	108.56 ± 25.68^c^	17.2 ± 3.3^c^	28.24 ± 5.66^c^

^a^Compared with control group *p* < 0.01.

^c^Compared with CCl_4_ group *p* < 0.01.

**Table 3:** Identification of the differential expressed proteins through the use of GO analysis.

Protein function categories	Up-regulation in rhCygb groups	Down-regulation in rhCygb groups
Response to stimulus		Haptoglobin; Adenine phosphoribosyltransferase
Oxidation-reduction process	Peroxiredoxin-2; Glutathione S-transferase Yb-3; Glutathione peroxidase 1; Cytochrome P450 2C11; Biliverdin reductase A; Estrogen sulfotransferase; Taste receptor type 1 member 2; Putative L-aspartate dehydrogenase	
Heat shock protein/chaperones		DnaJ homolog subfamily B member 1; GTP-binding protein SAR1b
Catabolic process	Long-chain-fatty-acid–CoA ligase 1; Enoyl-CoA hydratase, mitochondrial; Carboxylesterase 1D; ATP synthase subunit alpha; Major urinary protein; Protein Inmt; Phenazine biosynthesis-like domain-containing protein; Ketohexokinase	
Cytoskeleton		Tubulin beta-4B chain; Microtubule-actin cross-linking factor 1; Cathepsin D

**Table 4:** GSEA result for the up-regulated genes in rhCygb treated groups vs CCl_4_ model group.

Gene Set Name	Description	Genes in Overlap	p-value	FDR q-value
SHETH LIVER CANCER VS TXNIP LOSS PAM4	Cluster PAM4: genes down-regulated in hepatocellular carcinoma (HCC) vs normal liver tissue from mice deficient for TXNIP [GeneID = 10628].	5	3.33 e^−8^	1.13 e^−4^
AMBROSINI FLAVOPIRIDOL TREATMENT TP53	Genes down-regulated by flavopiridol [PubChem = 5287969] in the HCT116 cells (colon cancer) depending on their TP53 [GeneID = 7157] status: wild-type vs loss of the gene’s function (LOF).	3	2.57 e^−5^	2.03 e^−2^
ACEVEDO_LIVER_CANCER_DN	Genes down-regulated in hepatocellular carcinoma (HCC) compared to normal liver samples	4	3.97 e^−5^	2.03 e^−2^
LEE LIVER CANCER SURVIVAL UP	Genes highly expressed in hepatocellular carcinoma with good survival.	3	8.05 e^−5^	3.39 e^−2^
HOUSTIS ROS	Genes known to modulate ROS or whose expression changes in response to ROS	2	8.98 e^−5^	3.39 e^−2^
VARELA ZMPSTE24 TARGETS DN	Top genes down-regulated in liver tissue from mice with knockout of ZMPSTE24 [GeneID = 10269].	2	8.98 e^−5^	3.39 e^−2^
OHGUCHI LIVER HNF4A TARGETS UP	Genes up-regulated in liver samples of liver-specific knockout of HNF4A [GeneID = 3172]	2	1.21 e^−4^	4.1 e^−2^
