# Supplementary material for: The Effect of rhCygb on CCl4-Induced Hepatic Fibrogenesis in Rat
Source: Sci Rep. 2016 Mar 23;6:23508. doi: 10.1038/srep23508 (PMC4804332; doi:10.1038/srep23508)
Supplement: Supplementary Information [file srep23508-s1.doc]

**Title page**

**Title:** **The Effect of rhCygb on CCl_4_-Induced Hepatic Fibrogenesis in Rat**

Zhen Li^1#^, Wei Wei^1#^, Bohong Chen^1#^, Gaotai Cai^1^, Xin Li^1^, Ping Wang^1^, Jinping Tang^1^, Wenqi Dong^1^*

^1^School of Biotechnology, Southern Medical University, Guangzhou, Guangdong Province 510515, PR China.

^#^ These authors contributed equally to this article. (Zhen Li^1#^, Wei Wei^1#^, Bohong Chen^1#^)

***Contact Information of Corresponding Author:**

Prof. Wenqi Dong

School of Biotechnology, Southern Medical University, Guangzhou, Guangdong Province 510515, PR China.

Tel: +86 20 61648556 Fax: +86 20 61648556

E-mail address: [dongwq63@263.net](mailto:dongwq63@263.net)(W. Dong).


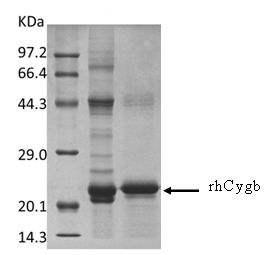


**Dataset 1. Purity detection of rhCygb**

**Dataset 2 Measurement of rhCygb anti-oxidant activity**

| sample | protein concentration  (mg/mL) | antioxidant activity  (U/mg) |
| --- | --- | --- |
| Control | 2 | 1.23±0.78 |
| rhCygb | 2 | 95.58±2.67^***^ |

^***^p<0.01


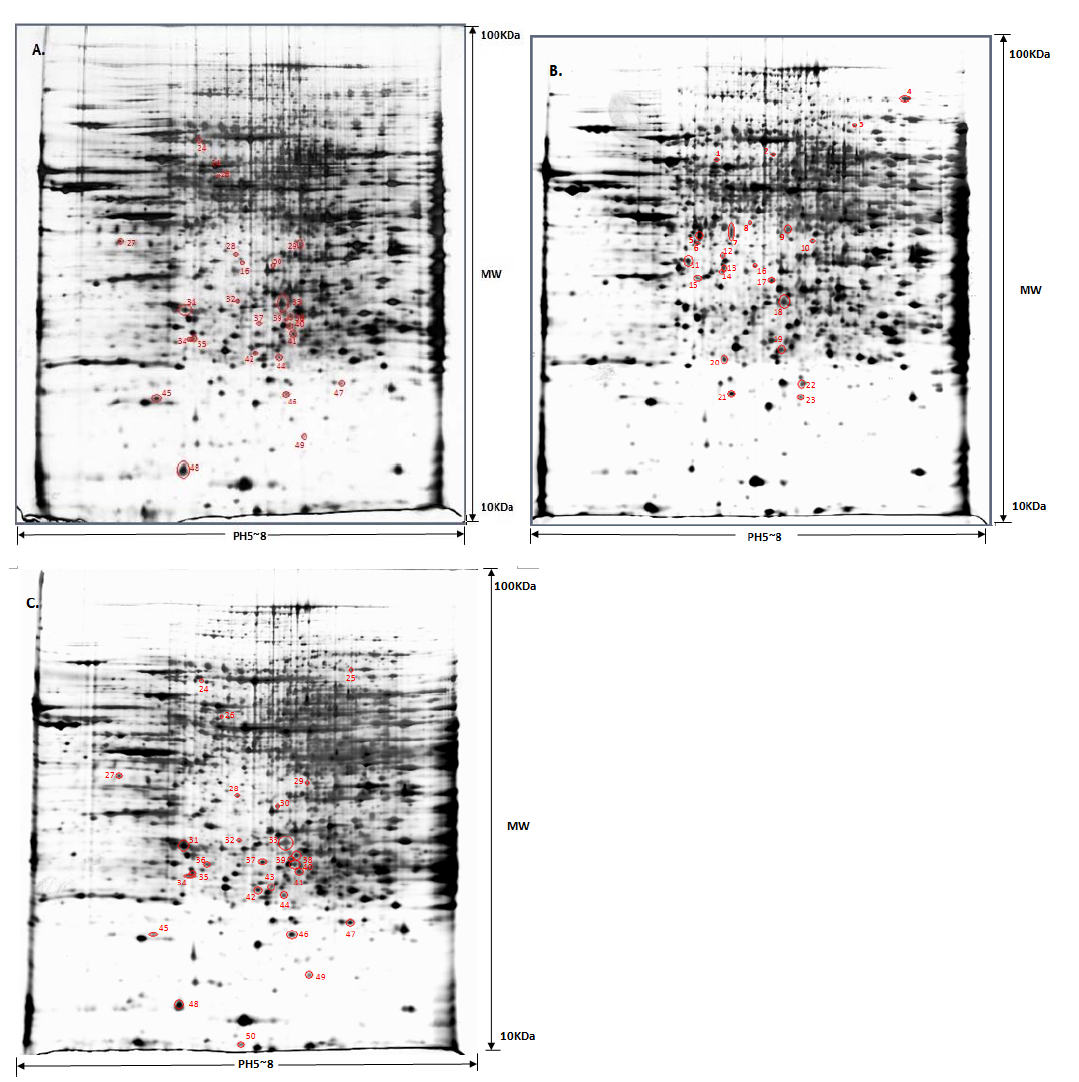


**Dataset 3. Differential proteins spots of rat liver proteins (A vehicle; B model; C rhCygb) on 2-DE gel.** First dimensional separation was performed on an immobilized nonlinear pH 5–8 strip, followed by the second-dimensional-separation on 12% SDS-PAGE. The protein spots were visualized by silver staining. The protein spots showed significantly different expression between A and B or B and C by image analysis and comparison and were marked with red circles and red ID codes.

**Dataset 4. GSEA result for the up-regulated genes in rhCygb treated groups vs CCl_4_ model groups**

**Dataset 5. Liver fibrosis scores of 150 rats by Metavir score system**

|  |  |  | LIVER HISTOLOGY ACTIVITY INDEX  (Metavir score system) | | | | |  |  |
| --- | --- | --- | --- | --- | --- | --- | --- | --- | --- |
| Group | Captivity | Rat | F0 | F1 | F2 | F3 | F4 | Sum | Mean Rank |
| Control(10+12wk) | 1 | 1 | √ |  |  |  |  | 10 | 17.5 |
|  |  | 2 | √ |  |  |  |  |  |  |
|  |  | 3 | √ |  |  |  |  |  |  |
|  |  | 4 | √ |  |  |  |  |  |  |
|  |  | 5 | √ |  |  |  |  |  |  |
|  | 2 | 1 | √ |  |  |  |  |  |  |
|  |  | 2 | √ |  |  |  |  |  |  |
|  |  | 3 | √ |  |  |  |  |  |  |
|  |  | 4 | √ |  |  |  |  |  |  |
|  |  | 5 | √ |  |  |  |  |  |  |
| CCl_4_  (10+12wk) | 3 | 1 |  |  |  |  | √ | 10 | 45.00 |
|  |  | 2 |  | √ |  |  |  |  |  |
|  |  | 3 |  |  |  |  | √ |  |  |
|  |  | 4 |  |  |  |  | √ |  |  |
|  |  | 5 |  |  |  |  | √ |  |  |
|  | 4 | 1 |  |  | √ |  |  |  |  |
|  |  | 2 |  |  |  |  | √ |  |  |
|  |  | 3 |  |  |  | √ |  |  |  |
|  |  | 4 |  |  |  |  | √ |  |  |
|  |  | 5 |  |  |  |  | √ |  |  |
| CCl_4_+  rhCygb  (10+12wk) | 5 | 1 | √ |  |  |  |  | 30 | 21.67 |
|  |  | 2 | √ |  |  |  |  |  |  |
|  |  | 3 | √ |  |  |  |  |  |  |
|  |  | 4 | √ |  |  |  |  |  |  |
|  |  | 5 |  | √ |  |  |  |  |  |
|  | 6 | 1 |  |  | √ |  |  |  |  |
|  |  | 2 | √ |  |  |  |  |  |  |
|  |  | 3 | √ |  |  |  |  |  |  |
|  |  | 4 | √ |  |  |  |  |  |  |
|  |  | 5 | √ |  |  |  |  |  |  |
|  | 7 | 1 | √ |  |  |  |  |  |  |
|  |  | 2 | √ |  |  |  |  |  |  |
|  |  | 3 |  |  | √ |  |  |  |  |
|  |  | 4 |  | √ |  |  |  |  |  |
|  |  | 5 | √ |  |  |  |  |  |  |
|  | 8 | 1 | √ |  |  |  |  |  |  |
|  |  | 2 |  | √ |  |  |  |  |  |
|  |  | 3 | √ |  |  |  |  |  |  |
|  |  | 4 | √ |  |  |  |  |  |  |
|  |  | 5 | √ |  |  |  |  |  |  |
|  | 9 | 1 | √ |  |  |  |  |  |  |
|  |  | 2 |  | √ |  |  |  |  |  |
|  |  | 3 | √ |  |  |  |  |  |  |
|  |  | 4 | √ |  |  |  |  |  |  |
|  |  | 5 | √ |  |  |  |  |  |  |
|  | 10 | 1 | √ |  |  |  |  |  |  |
|  |  | 2 | √ |  |  |  |  |  |  |
|  |  | 3 | √ |  |  |  |  |  |  |
|  |  | 4 | √ |  |  |  |  |  |  |
|  |  | 5 | √ |  |  |  |  |  |  |
| **10+12wk**  Kruskal-Wallis Test：*p*=0.000  Control vs CCl_4_：*p* =0.000  Control vs CCl_4_+rhCygb：*p* =0.131  CCl_4_ vs CCl_4_+rhCygb：*p* =0.000 | | | | | | | | | |
| Control(13+12wk) | 11 | 1 | √ |  |  |  |  | 10 | 13.50 |
|  |  | 2 | √ |  |  |  |  |  |  |
|  |  | 3 | √ |  |  |  |  |  |  |
|  |  | 4 | √ |  |  |  |  |  |  |
|  |  | 5 | √ |  |  |  |  |  |  |
|  | 12 | 1 | √ |  |  |  |  |  |  |
|  |  | 2 | √ |  |  |  |  |  |  |
|  |  | 3 | √ |  |  |  |  |  |  |
|  |  | 4 | √ |  |  |  |  |  |  |
|  |  | 5 | √ |  |  |  |  |  |  |
| CCl_4_  (13+12wk) | 13 | 1 |  |  |  | √ |  | 10 | 45.20 |
|  |  | 2 |  |  |  |  | √ |  |  |
|  |  | 3 |  |  |  |  | √ |  |  |
|  |  | 4 |  |  |  |  | √ |  |  |
|  |  | 5 |  |  |  |  | √ |  |  |
|  | 14 | 1 |  |  |  |  | √ |  |  |
|  |  | 2 |  |  |  |  | √ |  |  |
|  |  | 3 |  |  |  | √ |  |  |  |
|  |  | 4 |  |  |  |  | √ |  |  |
|  |  | 5 |  |  |  |  | √ |  |  |
| CCl_4_+  rhCygb  (13+12wk) | 15 | 1 |  |  |  | √ |  | 30 | 22.93 |
|  |  | 2 | √ |  |  |  |  |  |  |
|  |  | 3 | √ |  |  |  |  |  |  |
|  |  | 4 |  | √ |  |  |  |  |  |
|  |  | 5 |  |  | √ |  |  |  |  |
|  | 16 | 1 |  |  | √ |  |  |  |  |
|  |  | 2 | √ |  |  |  |  |  |  |
|  |  | 3 | √ |  |  |  |  |  |  |
|  |  | 4 | √ |  |  |  |  |  |  |
|  |  | 5 | √ |  |  |  |  |  |  |
|  | 17 | 1 |  | √ |  |  |  |  |  |
|  |  | 2 |  | √ |  |  |  |  |  |
|  |  | 3 | √ |  |  |  |  |  |  |
|  |  | 4 | √ |  |  |  |  |  |  |
|  |  | 5 | √ |  |  |  |  |  |  |
|  | 18 | 1 |  |  | √ |  |  |  |  |
|  |  | 2 | √ |  |  |  |  |  |  |
|  |  | 3 |  |  |  | √ |  |  |  |
|  |  | 4 |  | √ |  |  |  |  |  |
|  |  | 5 | √ |  |  |  |  |  |  |
|  | 19 | 1 | √ |  |  |  |  |  |  |
|  |  | 2 |  | √ |  |  |  |  |  |
|  |  | 3 | √ |  |  |  |  |  |  |
|  |  | 4 |  |  | √ |  |  |  |  |
|  |  | 5 | √ |  |  |  |  |  |  |
|  | 20 | 1 | √ |  |  |  |  |  |  |
|  |  | 2 |  |  |  | √ |  |  |  |
|  |  | 3 | √ |  |  |  |  |  |  |
|  |  | 4 |  | √ |  |  |  |  |  |
|  |  | 5 |  | √ |  |  |  |  |  |
| **13+12wk**  Kruskal-Wallis Test：*p* =0.000  Control vs CCl_4_：*p* =0.000  Control vs CCl_4_+rhCygb：*p* =0.010  CCl_4_ vs CCl_4_+rhCygb：*p* =0.000 | | | | | | | | | |
| Control(15+12wk) | 21 | 1 | √ |  |  |  |  | 10 | 15.00 |
|  |  | 2 | √ |  |  |  |  |  |  |
|  |  | 3 | √ |  |  |  |  |  |  |
|  |  | 4 | √ |  |  |  |  |  |  |
|  |  | 5 | √ |  |  |  |  |  |  |
|  | 22 | 1 | √ |  |  |  |  |  |  |
|  |  | 2 | √ |  |  |  |  |  |  |
|  |  | 3 | √ |  |  |  |  |  |  |
|  |  | 4 | √ |  |  |  |  |  |  |
|  |  | 5 | √ |  |  |  |  |  |  |
| CCl_4_  (15+12wk) | 23 | 1 |  |  |  |  | √ | 10 | 45.40 |
|  |  | 2 |  |  |  |  | √ |  |  |
|  |  | 3 |  |  |  |  | √ |  |  |
|  |  | 4 |  |  |  |  | √ |  |  |
|  |  | 5 |  |  |  |  | √ |  |  |
|  | 24 | 1 |  |  |  |  | √ |  |  |
|  |  | 2 |  |  |  |  | √ |  |  |
|  |  | 3 |  |  |  |  | √ |  |  |
|  |  | 4 |  |  |  | √ |  |  |  |
|  |  | 5 |  |  |  |  | √ |  |  |
| CCl_4_+  rhCygb  (15+12wk) | 25 | 1 |  | √ |  |  |  | 30 | 22.37 |
|  |  | 2 |  | √ |  |  |  |  |  |
|  |  | 3 | √ |  |  |  |  |  |  |
|  |  | 4 | √ |  |  |  |  |  |  |
|  |  | 5 | √ |  |  |  |  |  |  |
|  | 26 | 1 | √ |  |  |  |  |  |  |
|  |  | 2 |  |  | √ |  |  |  |  |
|  |  | 3 | √ |  |  |  |  |  |  |
|  |  | 4 | √ |  |  |  |  |  |  |
|  |  | 5 |  |  |  | √ |  |  |  |
|  | 27 | 1 | √ |  |  |  |  |  |  |
|  |  | 2 | √ |  |  |  |  |  |  |
|  |  | 3 |  | √ |  |  |  |  |  |
|  |  | 4 |  | √ |  |  |  |  |  |
|  |  | 5 | √ |  |  |  |  |  |  |
|  | 28 | 1 | √ |  |  |  |  |  |  |
|  |  | 2 |  |  | √ |  |  |  |  |
|  |  | 3 | √ |  |  |  |  |  |  |
|  |  | 4 | √ |  |  |  |  |  |  |
|  |  | 5 | √ |  |  |  |  |  |  |
|  | 29 | 1 | √ |  |  |  |  |  |  |
|  |  | 2 |  | √ |  |  |  |  |  |
|  |  | 3 | √ |  |  |  |  |  |  |
|  |  | 4 | √ |  |  |  |  |  |  |
|  |  | 5 |  |  |  | √ |  |  |  |
|  | 30 | 1 | √ |  |  |  |  |  |  |
|  |  | 2 | √ |  |  |  |  |  |  |
|  |  | 3 |  | √ |  |  |  |  |  |
|  |  | 4 |  | √ |  |  |  |  |  |
|  |  | 5 | √ |  |  |  |  |  |  |
| **15+12wk**  Kruskal-Wallis Test：*p* =0.000  Control vs CCl_4_： *p* =0.000  Control vs CCl_4_+rhCygb：*p* =0.028  CCl_4_ vs CCl_4_+rhCygb： *p* =0.000 | | | | | | | | | |
